# Pre-distance assessment from radial artery to lower extremity arterial lesion

**DOI:** 10.1007/s10554-025-03328-7

**Published:** 2025-01-08

**Authors:** Arata Sano, Takeshi Sugimoto, Tomoya Iwasaki, Tomonori Miki, Shigeki Takai, Noriyuki Wakana, Kan Zen, Hiroyuki Yamada, Satoaki Matoba

**Affiliations:** 1 Department of Cardiovascular Medicine, Kyoto Prefectural University of Medicine, Kyoto, Japan; 2 Department of Cardiovascular Medicine, Kyoto Tanabe Central Hospital, 6-1-6 Tanabe chuo, Kyotanabe-city, Kyoto, 610-0334 Japan; 3 Department of Radiology, Kyoto Tanabe Central Hospital, Kyotanabe, Japan

**Keywords:** Lower extremity artery disease, Endovascular treatment, Transradial artery intervention, Computed tomography

## Abstract

Endovascular treatment (EVT) for patients with lower extremity artery disease is widely used as a less invasive alternative to surgical bypass. Recently, transradial artery intervention has gained popularity owing to its minimally invasive nature. The distance from the radial artery to the target vessel is critical for success; however, effective pre-assessment methods have not yet been established. This study aimed to evaluate the usefulness of predistance measurements from the left radial artery using simple computed tomography (CT) images. In this study, distance measurements were performed from the left radial artery to the left and right iliac artery bifurcations and from the left radial artery to the common femoral artery at the upper femoral border. These distances, measured using CT images before and after the lower-extremity contrast study, were compared with the distances identified during the lower-extremity contrast study. Distances measured using simple CT images showed a high correlation with the distances identified during the lower-extremity contrast examination (*r* = 0.9317, *p* < 0.0001; from the left radial artery to the left and right iliac artery bifurcation; *r* = 0.9402, *p* < 0.0001; and from the left radial artery to the right common femoral artery at the upper femoral border). Our results suggest that pre-distance measurement using simple CT images can be a useful tool for EVT using the left radial artery approach. Although future large-scale studies are required, this technique merits consideration owing to its widespread adoption in clinical practice.

## Introduction

The prevalence of lower extremity artery disease (LEAD) has been on the rise in recent years, primarily due to an aging population and an increase in lifestyle-related diseases, often a consequence of dietary westernization [1, 2]. It significantly impacts patients’ quality of life, leading to pain, limited mobility, and, in severe cases, limb amputation. The increasing incidence of LEAD highlights the urgent need for effective management strategies [2].

Historically, surgical bypass was the standard treatment for severe cases of LEAD. However, surgical interventions are invasive, require longer recovery times, and pose higher risks, especially for older adults or those with comorbid conditions [3, 4]. In response to these challenges, endovascular treatment (EVT) has emerged as a less invasive alternative that offers comparable efficacy with reduced recovery times and lower complication rates [5].

The adoption of EVT has been steadily increasing globally and particularly in Japan, reflecting its growing importance in managing LEAD [6–8]. EVT can be performed using various access sites, each with unique advantages and disadvantages. The femoral artery has traditionally been the primary access point for these procedures. The femoral approach, though effective, is associated with a higher incidence of access site complications such as hematoma, pseudoaneurysm, and bleeding, particularly in patients on anticoagulant therapy or with coagulopathies [9, 10]. These complications can lead to prolonged hospital stays and increased healthcare costs, thus diminishing the overall benefits of the procedure.

Recent advances in vascular access techniques have led to a shift towards using the radial artery as an alternative access site. This change was motivated by the high incidence of complications associated with femoral artery puncture. The radial artery approach, initially popularized in the field of percutaneous coronary intervention (PCI), has been shown to significantly reduce access site complications [11]. In patients with chronic kidney disease, the radial artery approach was associated with a lower risk of in-hospital mortality, postprocedural bleeding, and blood transfusion than the femoral artery approach [12]. This trend is now extending to EVT, where the radial artery approach is increasingly recognized for its safety and efficacy [13]. Nowadays, EVT using the distal radial artery approach has been reported to be less invasive [14].

Furthermore, patient satisfaction with the radial artery approach was notably higher than with the femoral artery approach. Studies have shown that patients undergoing procedures via the radial artery report less discomfort and quicker post-procedure mobilization, contributing to an overall better patient experience [15].

However, despite these benefits, transradial intervention (TRI) has limitations owing to the constraints of the available medical devices. The distance from the radial artery to the target vessel plays a crucial role in the success of TRI. Effective pre-assessment methods for measuring this distance have not yet been established. Accurate measurement of the distance from the radial artery to the target vessel is crucial for selecting appropriate devices and planning the procedure.

Therefore, this study aimed to evaluate the effectiveness of simple Computer Tomography (CT) imaging in measuring the preliminary distance from the left radial artery to the target vessel. This study aimed to establish a reliable pre-assessment method to facilitate the increased adoption of the radial artery approach in EVT.

## Materials and methods

### Study cohort

**Fig. 1:**
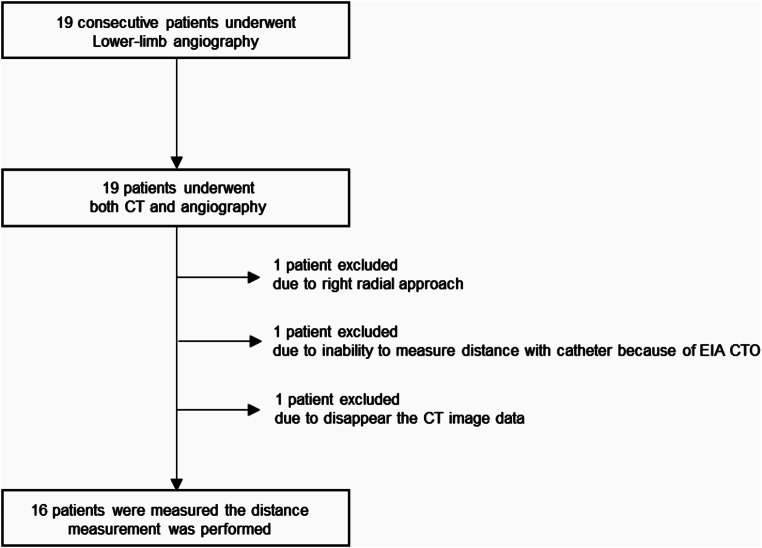
Flowchart of this study. 16 patients were enrolled in this study

This was a retrospective, single-center clinical investigation. Between January 2024 and June 2024, a total of 19 consecutive patients (10 men and 9 women) were enrolled in this study (Fig. 1). All patients had symptoms of intermittent claudication or chronic limb threatening ischemia, thus a catheter examination was performed for diagnosis. One patient was excluded due to the right radial approach, one patient was excluded due to the inability to measure the distance with the catheter due to chronic total occlusion in the external iliac artery, and one patient was excluded due to the disappearance of CT image data. 16 consecutive patients were enrolled in this study. All patients underwent lower limb angiography via the left radial or distal radial arteries. All patients underwent simple computed tomography (CT) with their hands down before and after lower limb angiography. This study was approved by the Ethics Committee of the Kyoto Tanabe Central Hospital (Approval No.: 2024-001).

### Computed tomography

The CT scan area ranged from the chest to below the knee. The distances from the left radial artery to the left and right iliac artery bifurcations, left radial artery to the right common femoral artery at the upper femoral border, and left radial artery to the left common femoral artery at the upper femoral border were measured.

CT images were acquired using an Aquilion ONE (Canon Medical Systems Corporation, Otawara, Tochigi, Japan). The detector collimation was 320 × 0.5 mm; the field of view was 500 mm; the gantry rotation speed was 0.5 s; the helical scanning; and the tube voltage was 80 kVp. An automatic exposure control with an SD of 8 was used for the tube current. Images were reconstructed with a slice thickness of 1.0 mm. All data were transferred and analyzed using Ziostation REVORAS (ZIOSOFT Inc, Tokyo, Japan).

#### Step.1.

A straight line is drawn from the three points of the proximal axillary artery (surgical neck level of the humerus) via the intersection of the radial and ulnar arteries, starting from the radial styloid process. (3 plots)

#### Step.2.

The axillary artery to the aortic arch is connected as short a distance as possible while following the vascular run. (15–20 plots)

#### Step.3.

The aortic arch to the iliac artery bifurcation is the shortest possible intravascular distance. (5–8 plots)

#### Step.4.

The iliac artery bifurcation to the popliteal artery is connected as short a distance as possible while following the vascular run.

Measure the distance between the lines connected by the above method.

### Catheter

#### Step.1.

After careful disinfection and local anesthesia, puncture the left radial artery and insert a sheath. The iliac artery contrast study is performed with a pigtail catheter. Radifocus Guide Wire M Standard Type (Terumo Corporation, Tokyo, Japan) was used to select the optimal artery, and if it is difficult, Radifocus Guide Wire M Stiff Type (Terumo Corporation) was used.

#### Step.2.

Replace the catheter with a 150 cm long multipurpose catheter and contrast the left and right superficial femoral arteries. This may be unilateral depending on renal function and lesion.

#### Step.3.

After contrasting the superficial femoral artery, withdraw the 150 cm-long multipurpose catheter up to the upper femoral border and measure the length of the extra multipurpose catheter outside the body from the radial styloid process.

Subtract the above from 150 cm to identify the distance from the left radial artery to the common femoral artery at the upper femoral border.

#### Step.4.

Withdraw the 150 cm-long multipurpose catheter to the iliac artery bifurcation and measure the length of the extra multipurpose catheter outside the body from the radial styloid process.

Subtract the above from 150 cm to determine the distance from the iliac artery bifurcation to the iliac artery bifurcation.

### Statistical analysis

All measurements were performed by a clinical technologist. Simple linear regression with Pearson’s correlation coefficient (r), coefficient of determination (R-squared), and p-values were calculated. Statistical significance was set at *p* < 0.05.

## Results

A total of 16 consecutive patients were enrolled in this study (Table 1). The average age was 78.4 years, with high proportion of dyslipidemia (88%) and chronic renal failure (75%). Two patients (13%) with critical limb ischemia were included, and all other patients had claudication symptoms. Also, over 80% of the patients took aspirin and P2Y12 inhibitors. The aortic arch type of the patients was showed as Type 1: 6%, Type 2: 56%, and Type 3: 38%, and no patients with bovine arch was observed. All patients underwent CT and angiography, and the distances from the left radial artery to the left and right iliac artery bifurcations, from the left radial artery to the right common femoral artery at the upper femoral border, and from the left radial artery to the left common femoral artery at the upper femoral border were measured and compared. In one case (Case 3), only the distance to the iliac artery bifurcation was measured because the catheter could not reach the bilateral common femoral artery at the upper femoral border. In three cases (Cases 1, 4, and 10), the distance was not measured on the healthy side owing to chronic renal function, and the distance was measured only on the affected side. Similarly, one patient (Case 2) did not measure the distance to the iliac artery bifurcation.

**Fig. 2:**
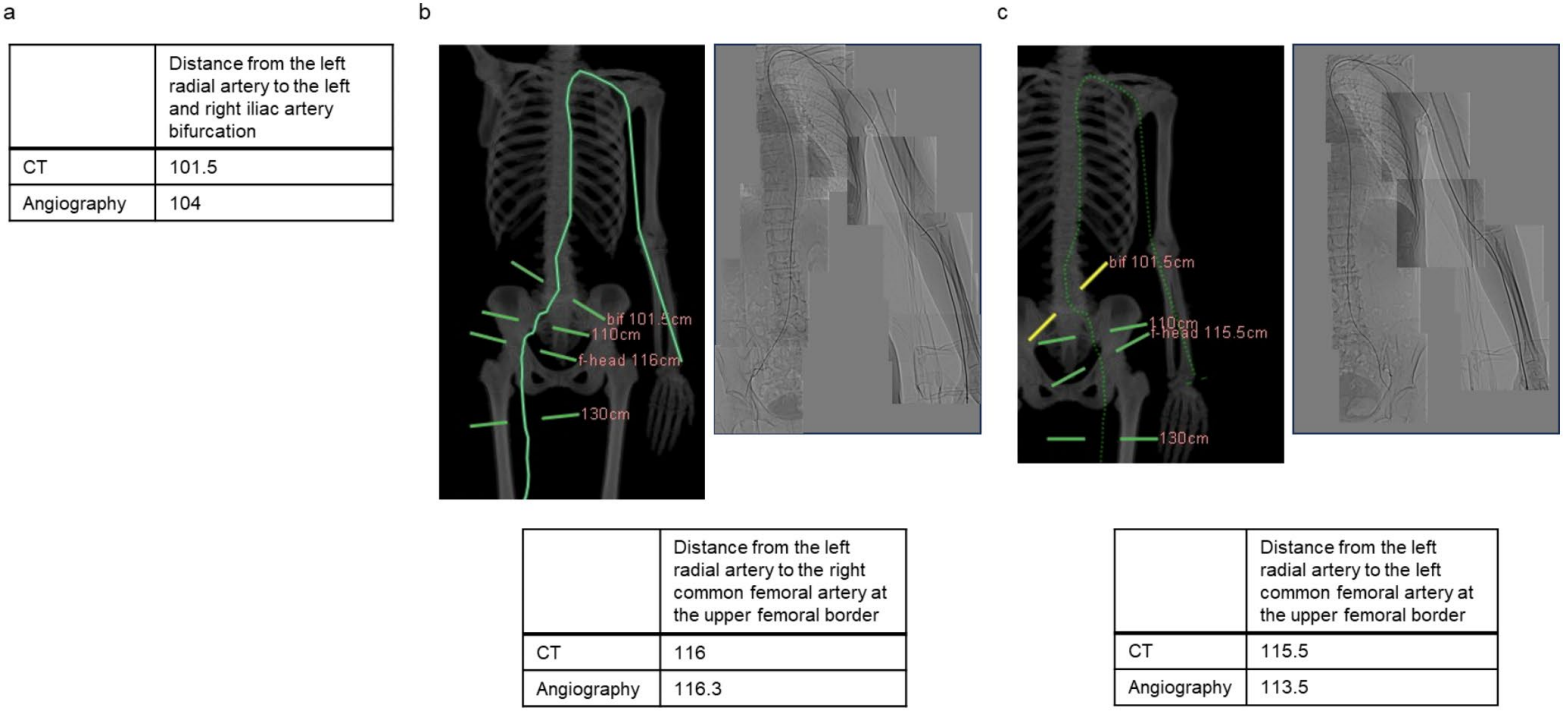
Representative case (Patient No. 15) showing: CT images (top-left in **b** and **c**), Angiography images (top-right in **b** and **c**), Measurements of them (top in **a**, bottom in **b** and **c**). (**a**) Distance from the left radial artery to the left and right iliac artery bifurcation. (**b**) Distance from the left radial artery to the right common femoral artery at the upper femoral border. (**c**) Distance from the left radial artery to the left common femoral artery at the upper femoral border

A representative case was that of a 65-year-old female (height: 154 cm) admitted with worsening intermittent claudication; thus, both CT and angiography were performed to confirm the diagnosis (Fig. 2a and b). Data from distance measurements are shown (Fig. 2c). A comparison between CT and angiographic measurements revealed that both sets of data were very similar.

**Table 1:**
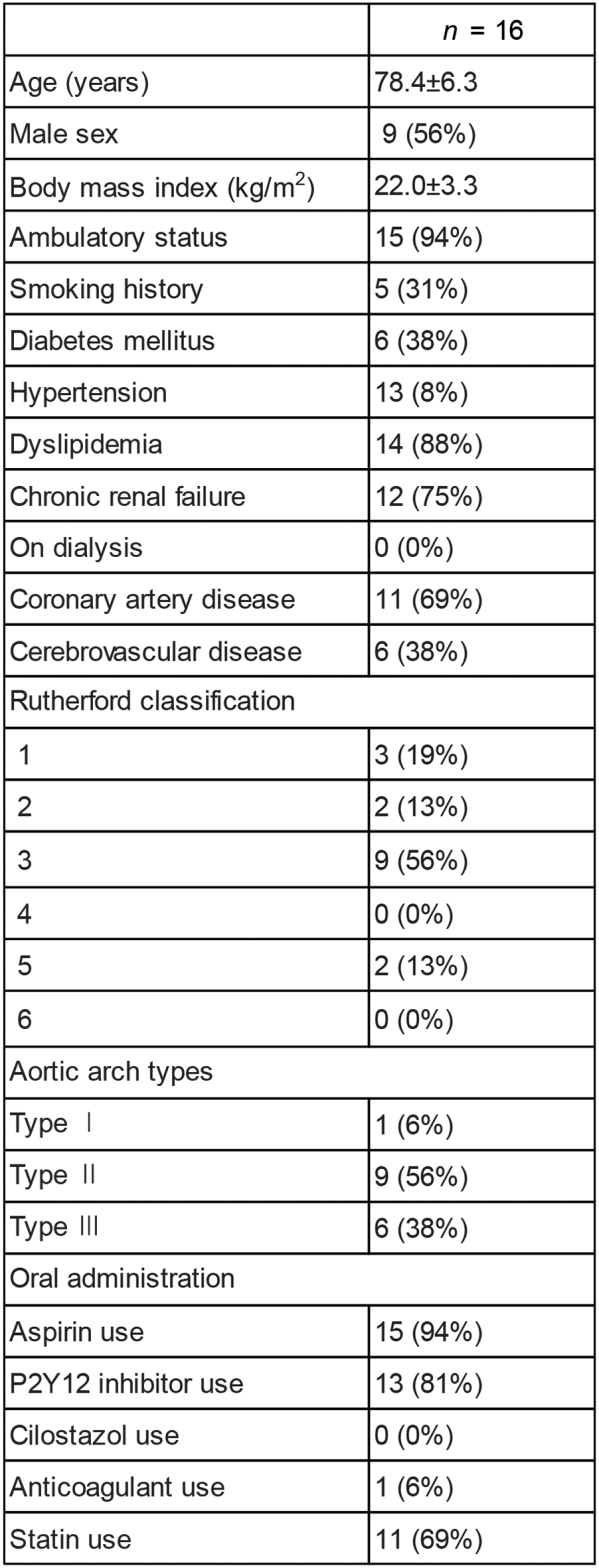
Characteristics of this study

**Table 2:**
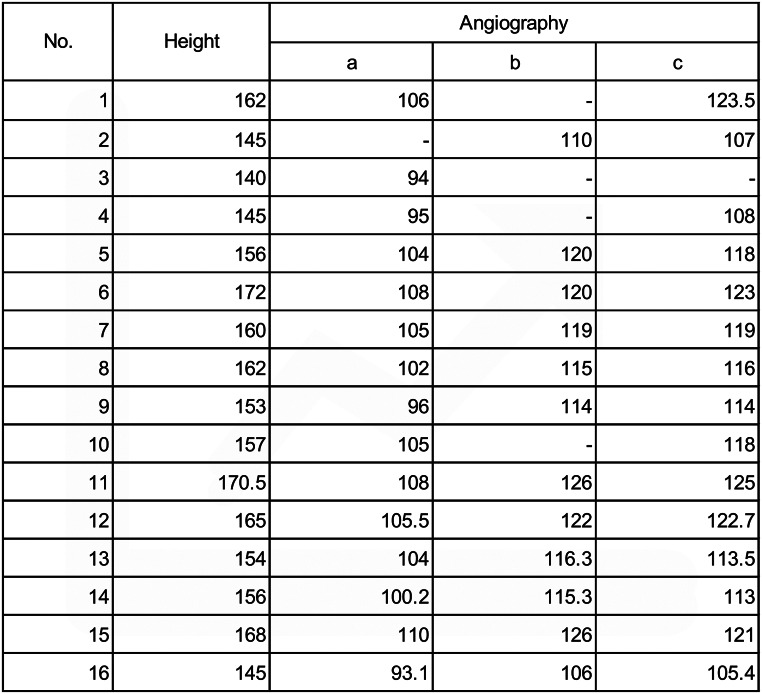
Comparison of patient height and distance measurements using angiography. (a) Distance from the left radial artery to the left and right iliac artery bifurcations. (b) Distance from the left radial artery to the right common femoral artery at the upper femoral border. (c) Distance from left radial artery to left common femoral artery at the upper femoral border

**Fig. 3:**
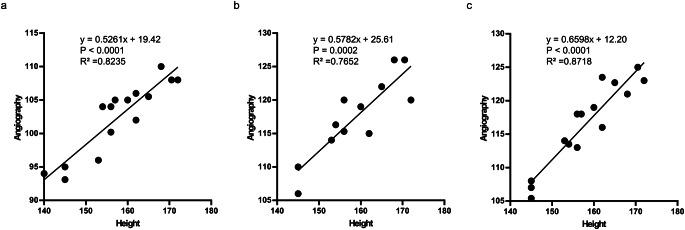
Distribution of patient height and distance measurements using angiography. (**a**) Distance from the left radial artery to the left and right iliac artery bifurcation. (**b**) Distance from the left radial artery to the right common femoral artery at the upper femoral border. (**c**) Distance from the left radial artery to the left common femoral artery at the upper femoral border

We first conducted an analysis to compare the height of patients with the angiographic measurement data of each patient. The heights of the patients and distance measurement data are shown (Table 2). A high correlation was obtained between the patient’s height and angiography measurement data (R² = 0.8235, *p* < 0.0001; from the left radial artery to the left and right iliac artery bifurcation), (R² = 0.7652, *p* = 0.0002; from the left radial artery to the right common femoral artery at the upper femoral border), and (R² = 0.8718, *p* < 0.0001; from the left radial artery to the left common femoral artery at the upper femoral border) (Fig. 3). The formula to predict the angiographic distance (y cm) from the height of the patient (x cm) was also obtained from each data point (y = 0.5261x + 19.42, from the left radial artery to the left and right iliac artery bifurcation), (y = 0.5782x + 25.61; from the left radial artery to the right common femoral artery at the upper femoral border), and (y = 0.6598x + 12.20; from the left radial artery to the left common femoral artery at the upper femoral border).

**Table 3:**
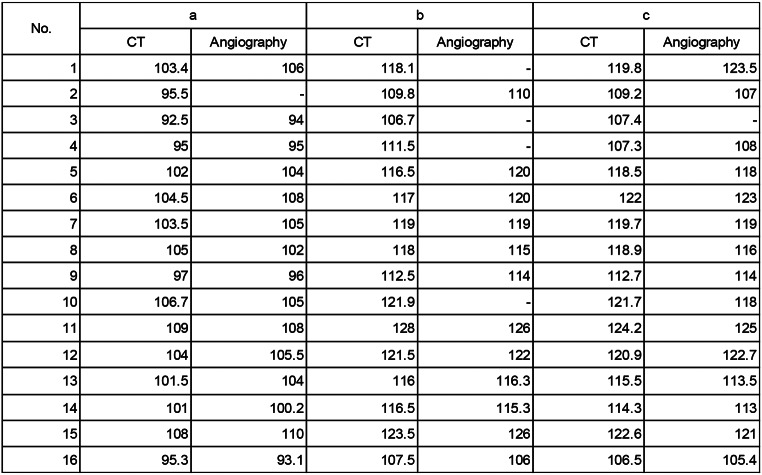
Comparision of distance measurement between CT and angiography. (a) Distance from the left radial artery to the left and right iliac artery bifurcations. (b) Distance from the left radial artery to the right common femoral artery at the upper femoral border. (c) Distance from left radial artery to left common femoral artery at the upper femoral border

**Fig. 4:**
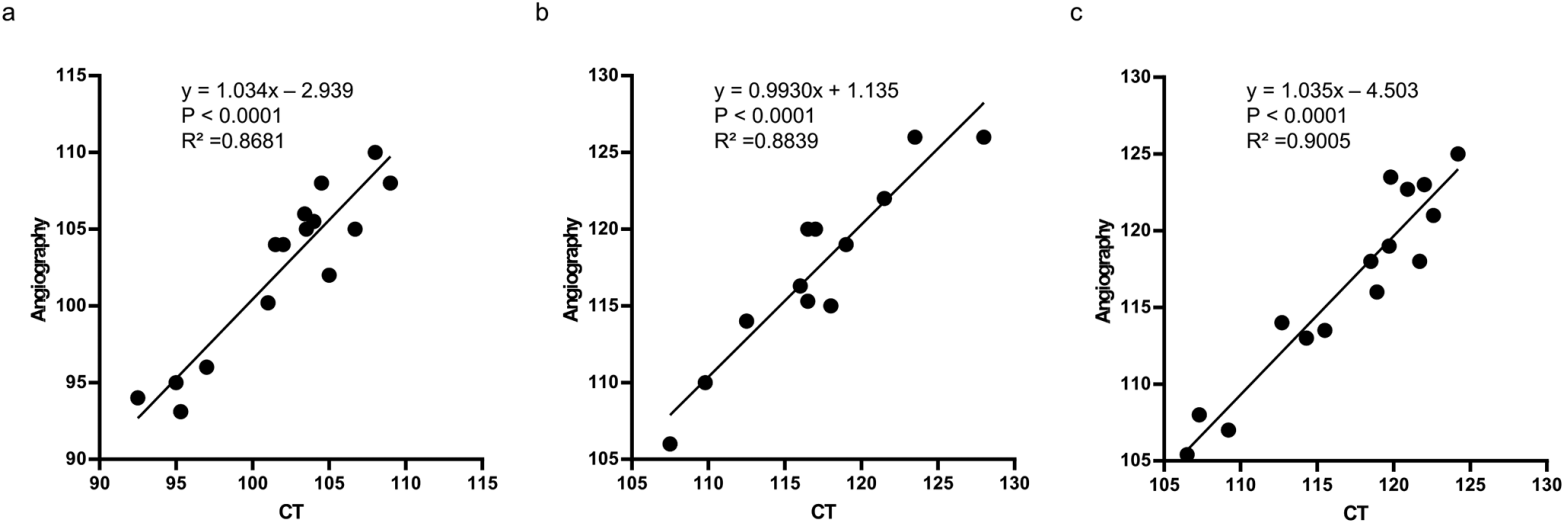
Distribution of distance measurement between CT and angiography. (**a**) Distance from the left radial artery to the left and right iliac artery bifurcation. (**b**) Distance from the left radial artery to the right common femoral artery at the upper femoral border. (**c**) Distance from the left radial artery to the left common femoral artery at the upper femoral border

Next, we performed a comparative analysis of CT and angiography. All measurement data for each patient are presented (Table 3). The accuracy of CT and angiography were compared. There were much higher concentrations between the two modalities (R² = 0.8681, *p* < 0.0001; from the left radial artery to the left and right iliac artery bifurcation), (R² = 0.8839, *p* < 0.0001; from the left radial artery to the right common femoral artery at the upper femoral border), and (R² = 0.9005, *p* < 0.0001; from the left radial artery to the left common femoral artery at the upper femoral border) (Fig. 4). The formula to expect the angiographical distance (y cm) from the distance measured by CT (x cm) was also obtained from each data (y = 1.034x – 2.939, from the left radial artery to the left and right iliac artery bifurcation), (y = 0.9930x + 1.135; from the left radial artery to the right common femoral artery at the upper femoral border), and (y = 1.035x – 4.503; from the left radial artery to the left common femoral artery at the upper femoral border).

**Fig. 5:**
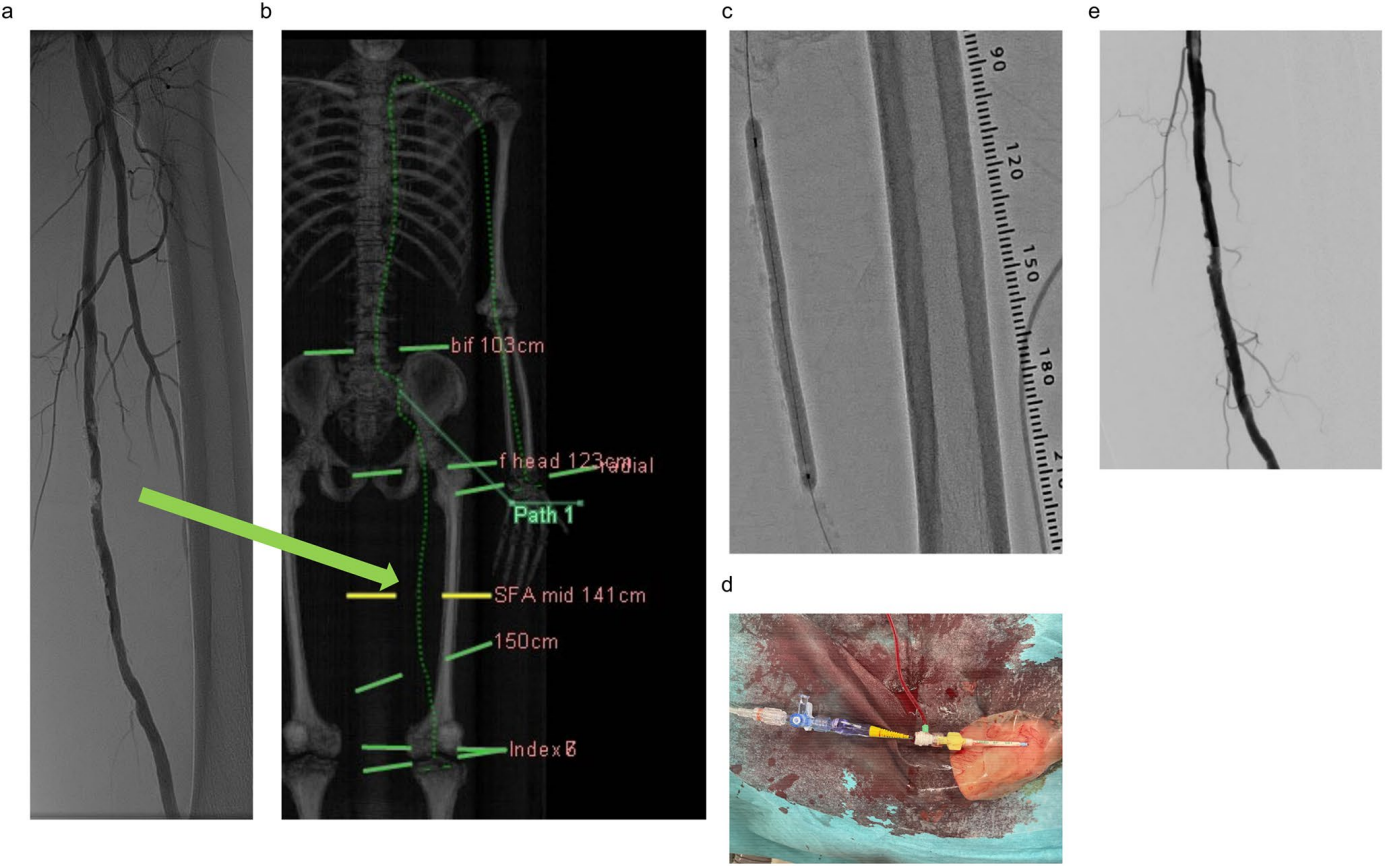
A case in which the puncture site was determined using preoperative CT. (**a**) Pre-operative angiographic image. (**b**) Pre-operative CT image. (**c**) Expanding DCB (Drug Coating Balloon) 5/100 cm. (**d**) Catheters outside the body. (**e**) Post-operative angiographic image

Because the above results suggested a very high correlation between CT and angiography, we prospectively performed a pre-operative review using CT. An 84-year-old man with arterial stenosis detected on lower extremity angiography was admitted to our hospital because of worsening symptoms of intermittent claudication (Fig. 5a). Preoperative CT revealed that the distance from the left radial artery to the site of stenosis was 141 cm (Fig. 5b). The total length was 149 cm, including a portion of the hemostatic valve outside the body. Thus, treatment via the left radial artery approach was feasible (Fig. 5c and d). This treatment was successful (Fig. 5e). The preoperative ABI of 0.69 improved to 0.95, and symptoms of intermittent claudication completely disappeared.

## Discussion

To our knowledge, this is the first study to demonstrate that distance measurements from the left radial artery using simple CT images are highly correlated with the distances identified during lower-extremity contrast examinations. These findings have significant implications for the pre-assessment and planning of EVT in patients with LEAD. By establishing a reliable pre-assessment method, we can potentially enhance procedural success and patient outcomes in EVT.

Currently, only a limited number of devices can be used from the radial artery. Stents with an effective length of 190 cm are available, whereas drug-coated balloons have the longest effective length of 150 cm. Guiding sheaths of various lengths are also available, but the choice limits the devices. Although new, more slender devices and balloon and stent delivery systems are being introduced to the market that may have sufficient length, the challenge of tortuous distal vessels and the ability to track these devices may limit the routine use of this technology for vascular surgeons, particularly with regard to the treatment of more distal lesions often seen in patients with chronic limb-threatening ischemia [13]. This study is more significant for its potential use in future trans-radial approaches for treating arteries below the inguinal ligament. Even if devices with longer effective lengths are developed in the future, preoperative distance measurements will allow the selection of a guiding sheath with an appropriate and easy-to-operate length, which will enable appropriate finalization. Accurate preprocedural measurements can enhance procedural planning, reduce intraprocedural complications, and improve overall outcomes in patients undergoing EVT.

Our findings suggest that CT, which is less invasive and more accessible, could serve as a superior modality for assessing the distance from the radial artery to the lesion to be treated. The advantages of CT imaging include its widespread availability, high-resolution imaging capabilities, and ability to provide comprehensive anatomical details. These features make it a valuable tool for pre-procedural planning in EVT. Additionally, CT can be performed relatively quickly and does not require the extensive preparation or recovery times associated with more invasive imaging techniques. In this study, 75% of the patients had chronic kidney disease, were not on maintenance dialysis, and could not undergo contrast CT imaging. In such cases, pre-assessment without contrast has great significance.

Furthermore, the safety of EVT with TRI has been demonstrated in a multicenter study [16]. Our study showed that preoperative CT imaging can be used to identify patients for whom TRI is feasible. In PCI, TRI has already been reported to be more economical than transfemoral intervention [17], and if this trend is also true for EVT, then our study can be synonymous with promoting optimal TRI for EVT, thus contributing to healthcare economics.

Although this study presents promising results, it is important to acknowledge its limitations. First, the sample size was relatively small and all patients were Japanese, which may have affected the generalizability of the findings. Also, the patients in this study were relatively short (140–172 cm), thus it is unclear whether the method used in this study is useful for the taller population. Second, the study was based on distance measurement using a multipurpose catheter rather than a guiding sheath. The guiding sheath is stiffer than the multipurpose catheter; therefore, it is assumed that the vessel will be stretched and straightened more than the multipurpose catheter. This could potentially lead to discrepancies in distance measurements, underscoring the need for further studies to validate these findings. Third, patients with aortic diseases such as aortic aneurysm or aortic dissection were not included in this study, which may have affected the data variety. Furthermore, in this study, we focused only on the left radial artery approach because it is easier to introduce the guiding sheath into the lower limb and the distance to the lower limb lesion is shorter. Further research is needed to determine whether similar results can be obtained using the right radial artery approach.

This study opens several avenues for future research. Large-scale studies should be conducted to confirm the reproducibility and reliability of CT-based distance measurements in various clinical settings. Additionally, the potential integration of this technique with real-time imaging modalities during EVT procedures could be explored to enhance intraprocedural guidance and precision. Real-time imaging techniques, such as intravascular ultrasound or optical coherence tomography, can be used in conjunction with preprocedural CT measurements to provide dynamic feedback during the procedure, further improving accuracy and outcomes. Preoperative imaging provides crucial anatomical data that can be incorporated into IoT (Internet of Things) networks to enhance precision during interventions, potentially improving treatment outcomes by aiding spatial orientation and minimizing risks in LEAD interventions [18].

## Conclusion

Our study suggests that distance measurement from the left radial artery using simple CT images is a reliable and effective pre-assessment tool for EVT in patients with LEAD. This technique has the potential to improve procedural planning, thereby improving patient outcomes. Considering the increasing use of the radial approach in endovascular procedures, the findings of this study are particularly relevant and warrant further investigation to support its widespread clinical adoption.

## Data Availability

No datasets were generated or analysed during the current study.

## Abbreviations

CT: Computer Tomography
EVT: Endovascular treatment
LEAD: Lower Extremity Artery Disease
PCI: Percutaneous coronary intervention
TRI: Transradial intervention
